# Sacituzumab govitecan: a promising antibody-drug conjugate for the treatment of poorly differentiated endometrial cancer

**DOI:** 10.18632/oncoscience.514

**Published:** 2020-06-08

**Authors:** Emily M. Webster, Burak Zeybek, Joan Tymon-Rosario, Alessandro D. Santin

**Affiliations:** ^1^Department of Obstetrics, Gynecology, and Reproductive Sciences, Division of Gynecologic Oncology, Yale School of Medicine, New Haven, CT, USA

**Keywords:** Sacituzumab govitecan, antibody-drug conjugate, endometrial cancer

Uterine cancer is the sixth most common malignancy in women worldwide, with the majority of cases originating from the endometrium [1]. The prognosis is generally favorable: an estimated two-thirds are diagnosed at an early stage, with a five-year survival rate of 78-90% for stage 1 and 74% for stage II [2, 3]. However, the five-year survival rate drops to 36-57% for stage III disease and 20% for stage IV [2]. Certain features portend a worse prognosis; Grade 3 (G3), or poorly differentiated endometrioid endometrial adenocarcinoma is associated with increased risk of advanced stage disease at time of diagnosis, increased rate of recurrence, and decreased survival [3, 4].

As with other types of endometrial cancer, the mainstay of treatment in women with G3 endometrioid adenocarcinoma is surgery including total hysterectomy, bilateral salpingo-oophorectomy, and staging. The optimal adjuvant treatment remains less defined, and National Comprehensive Cancer Network (NCCN) Guidelines offer several options for each stage [5]. Women with stage IA disease are recommended to undergo vaginal brachytherapy. External beam radiation therapy (EBRT) and observation are alterative options in the settings of high-intermediate risk features and no myoinvasion, respectively. Adjuvant treatment for those with stage IB and II consists of radiation therapy (EBRT and/or vaginal brachytherapy) with the optional addition of systemic therapy. For those with stage III/IV, advanced and recurrent disease, systemic therapy is essential. Regardless of initial treatment, recurrent and metastatic disease is associated with poor prognosis, as few options exist after first-line treatment.

Targeted therapy remains an area of exploration for development of new agents such as antibody-drug conjugates (ADCs), targeted monoclonal antibodies linked to a cytotoxic agent. Sacituzumab govitecan, an ADC that is comprised of a humanized anti-trophoblast cell surface antigen (Trop-2) antibody, conjugated with the active metabolite of irinotecan (SN-38) through the cleavable CL2A linker, has shown promising results against epithelial cancers overexpressing Trop-2 in Phase II trials [6-9]. Trop-2 is a transmembrane calcium signal transducer that is highly expressed by diverse epithelial solid tumors and has been shown to be a prognostic marker in many of these cancers including endometrioid endometrial carcinomas [10]. Strong Trop-2 immunostaining is significantly associated with higher tumor grade and reduced disease-free survival [10].

Perrone *et al.* recently investigated Trop-2 expression in 143 patients with G3 endometrioid endometrial adenocarcinomas and studied the cytotoxic effects of sacituzumab govitecan against these tumors in the preclinical setting [11]. Moderate to strong Trop-2 expression was identified on tissue microarray in 84% (120/143) of the patients. In an *in vitro* model, the authors demonstrate both antibody-dependent cellular toxicity as well as cytotoxic effects of sacituzumab govitecan in tumors expressing Trop-2. The same toxic effects were not seen in Trop-2 negative cell lines. The authors also noted a bystander effect, which is a unique characteristic of ADCs with cleavable linkers that provides an opportunity for destruction of not only the antigen-positive target cells but also the surrounding antigen-negative cells. This is especially beneficial in tumors that demonstrate high heterogeneity in the expression of target antigens such as uterine cancer. In the xenograft model, intravenous administration of sacituzumab govitecan twice weekly for a total of six injections resulted in a significant tumor growth inhibition.

These promising preclinical results strongly suggest that sacituzumab govitecan may represent a novel, potentially effective treatment option for poorly differentiated endometrial cancer patients with recurrent/metastatic disease resistant to standard treatment modalities. The results of the first clinical trial of trop-2 targeted therapy in uterine cancer (NCT04251416) are eagerly awaited and will shed more light into this topic.
